# Podoconiosis in Rural Tanzania

**DOI:** 10.4269/ajtmh.16-0028

**Published:** 2016-07-06

**Authors:** Ryan Eid, Dhruv Sharma, William Smock

**Affiliations:** ^1^ University of Louisville School of Medicine, Louisville, Kentucky.

**Figure 1.**
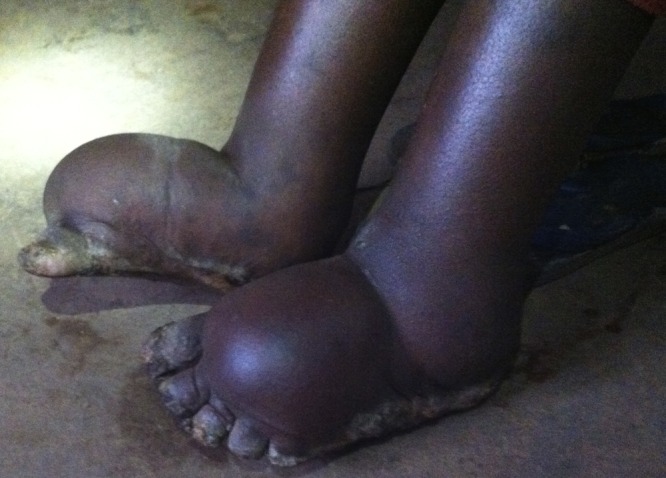
Initial presentation of podoconiosis with bilateral “water bag” edema and block-shaped toes.

A 30 year-old woman who lived in the southern highlands of Tanzania presented with a 5-year history of progressive bilateral foot and ankle swelling. Mossy-like papillomata and block-shaped toes involving both feet were apparent, and the swelling had the consistency of a “water-bag” (Figure 1

). The patient denied travel to Tanzania's coastal region. The symptoms of elephantiasis in the absence of exposure to areas where the mosquito vector for *Wuchereria bancrofti* is found make lymphatic filariasis unlikely and suggest podoconiosis.

Podoconiosis is known as “mossy-foot” because the papillomata have a moss-like appearance. It is caused by long-term barefoot exposure to volcanic soils high in silica. These soils are found in the highlands of tropical Africa, Central America, and northwest India.^1^ Seasonally heavy rains in these regions lead to soil erosion. Chronic, recurrent barefoot exposure to exposed silica leads to lymphatic obstruction resulting in ascending lymphedema.^2^

**Figure 2.**
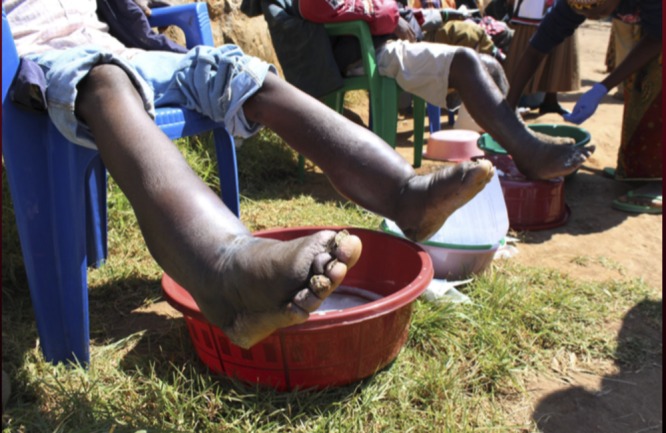
Podoconiosis with bilateral “water bag” edema. Note that the swelling terminates at the level of the knee joint.

Podoconiosis is clinically distinguished from filarial elephantiasis. Unlike filarial elephantiasis, podoconiosis characteristically presents with block-shaped toes, mossy-like papillomata, and an ascending edema that stops at the knee without groin involvement (Figure 2

). It is important to recognize that the presentation of podoconiosis can vary from the classic hard, nodular edema to a soft “water-bag” edema.^1^

Podoconiosis is preventable with consistent shoe wearing and good foot hygiene practices. Treatment includes use of compression bandages and elevation of the affected limbs.^3^ With time and proper treatment the edema and moss-like papillomata can diminish (Figure 2).
